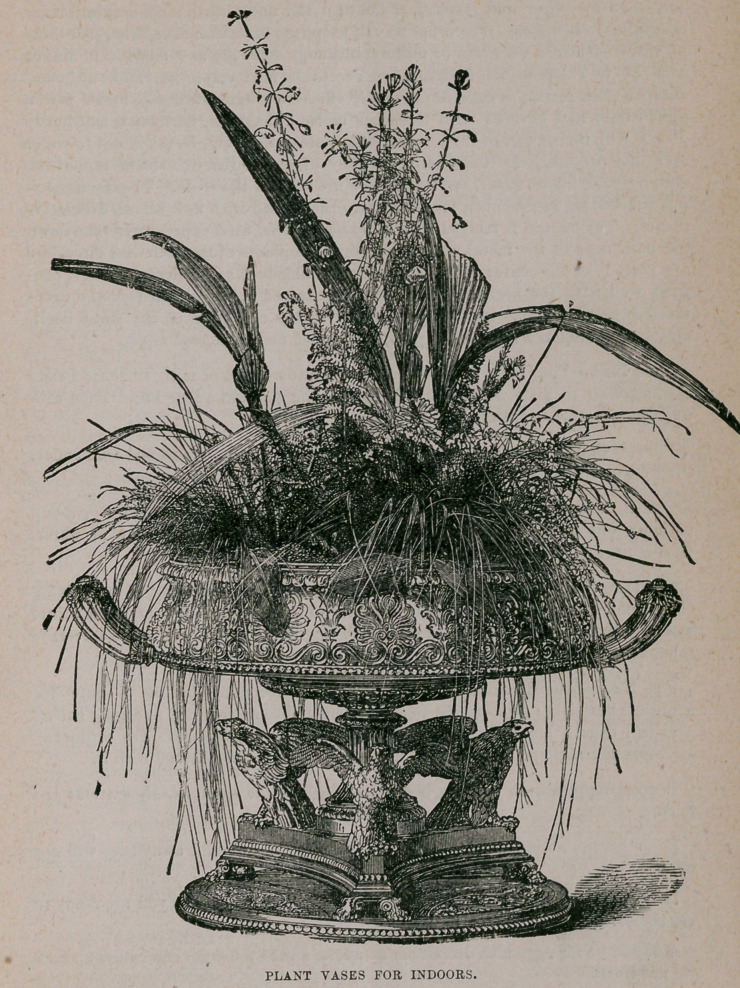# Household

**Published:** 1888-09

**Authors:** 


### HOUSEHOLD.

PLANT VASES FOR INDOORS.

### PLANT VASES FOR INDOORS.

The votaries of floriculture are now turning their attention indoors, and inquiries as to proper and tasteful modes of parlor and dinner table decoration are beginning to be made. The usual way of keeping plants in houses is to place them in vases or tazzas of wood or pottery, although some are now made in bronze or iron, of very handsome designs ; terra-cotta is also employed, and, although cheaper than metal, is capable of equally effective ornamentation. Filled with a light earth, and covered with the moss called Sphagnum, hardy and half hardy plants will thrive well in these vases. Care must, however, be taken not to water them too profusely, as there being no way of escape through the bottom of the vase for superfluous water, too much moisture will rot the roots. The accompanying illustration represents a bronze tazza, ornamented with well known decorative plants. The margin is fringed with *isolepsis gracilis*, used expressly to tone down the harshness of the metal work. Two or three plants of the palm-like circuiligo are placed in the center, and these, by furnishing bold and graceful foliage, contract well with the horizontal lines of the tazza below, while their cool and deep-toned greenness forms a pleasing contrast to the character of the stand itself. Heaths and similar hard-wooded plants are added and with good effect.

Rice Pies.—Four eggs, well beaten, stirred into a quart of milk, two cups boiled rice, sweeten to taste and flavor. When boiling rice add a litte salt. Bake with under crust same as custard pies.

Coffee Cake.—One cup each of sugar, molasses, butter, raisins and cold coffee, three cups of flour, one teaspoonful of soda and two of cinnamon.

Cream Sponge Cake.—Two eggs, one-half cup of sugar, three-fourths or a cup of flour, one teaspoonful of baking powder, two tablespoonfuls of cold water. Beat white and yelks separately.

To Make Paper Stick to Whitewashed Walls.—A writer says: "Make a sizing of common glue and water, of the consistency of linseed oil, and apply with white-wask or other brush to the wall, taking care to go over every part, and especially, top and bottom. Apply the paper in the ordinary way.

For Neuralgic Headache.—The following is recommended as a cure for neuralgic headache: Squeeze the juice of a lemon into a small cup of strong coffee. This will usually afford immediate relief in neuralgic headache. Tea ordinarily increases neuralgic pain, and ought not be used by persons affected with it.

In roasting meats do not salt before putting into the oven, as salt extracts the juice.

If clothes are absolutely dry before they are folded and laid away they will not mildew.

If warm weather put your eggs in cold water some time before you are ready to use them.

A true test for eggs is to drop them in water, and if the large end comes up they are not fresh.

## Figures and Tables

**Figure f1:**